# News and Coming Events

**Published:** 1909-07-24

**Authors:** 


### NEWS AND COMING EVENTS.

Dr. Isaac Mossop has been appointed by the Lord Chancellor to the Commission of the Peace for the City of Bradford.

Mr. F. N. Doubleday, L.D.S., R.C.S.(Eng.), has been awarded the Guy's Hospital Travelling Scholarship of the value of £100.

The University of Geneva has recently conferred upon Lord Lister the honorary degree of Doctor of Medicine in recognition and appreciation of his epoch-making contributions to surgical science and practice.

The Lord Lieutenant of Bedfordshire has appointed Surgeon-Lieut.-Colonel Rowland Hill Coombs, M.D., of the 3rd battalion the Bedfordshire Regiment (Special Reserve), to the post of Deputy Lieutenant of the County.

Professor William Stirling, M.D., Dean of the Medical Faculty, pro Vice-Chancellor of the University, and Professor of Physiology in Manchester University, has been nominated to represent the University as its official delegate at the forthcoming quincentenary of the University of Leipsic, to be celebrated from July 28 to 31.

A General Court of Governors of the East London Hospital for Children, and Dispensary for Women, Shadwell, E., will be held at the Hospital on Monday, July 26, at 4 o'clock in the afternoon, to receive a report on the general state of the institution, from the Board of Management, and transact other business.

The following awards of Fellowships have been made in connection with the recent degree examinations at the University of Liverpool:—Robert Gee Fellowship in Anatomy, R. H. Mole, M.D.; Holt Fellowship in Physiology, C. H. H. Harold, M.B., Ch.B.; Thelwall Thomas Fellowship, W. W. Mackarell, M.B., Ch.B.; Ethel Boyce Fellowship, W. R. Pierce, M.B., Ch.B.

The honorary degree of M.D. of the University of Dublin has been conferred upon the following eminent English and Scottish medical men:—Sir Alfred Keogh, M.D., K.C.B., Director-General of the Army Medical Service; Dr. W. Hale White, senior physician to Guy's Hospital; Dr. W. P. Herringham, physician to St. Bartholomew's Hospital; Dr. G. A. Gibson, physician to the Royal Infirmary, Edinburgh; and Dr. G. Redmayne Murray, Professor of Pathology in Victoria University, Manchester.

Royal Army Medical College Prizes.—The War Office has issued the following list of prize-winners at the recent examination at the termination of the junior course at the Royal Army Medical College:—Lieut. H. S. Ranken, R.A.M.C., the Herbert, De Chaumont, Tulloch Memorial, Ranald Martin, and Marshall Webb prizes; Lieut. J. A. Manifold, R.A.M.C., the 1st Montefiore prize; Lieut. C. L. Franklin, R.A.M.C., the 2nd Montefiore prize; Lieut. A. M. S. Jukes, I.M.S., the Parkes Memorial prize; and Lieut. B. Gale, I.M.S., the Fayrer Memorial prize.

The following appointments have been made to the medical staff of St. John's Hospital for Diseases of the Skin, Leicester Square, London, W.C. :—Dr. William Griffith, M.B., Ch.B. Victoria, M.R.C.P. Lond., has been appointed Assistant Physician; Dr. C. A. McBride, M.D., C.M. Toronto, L.R.C.P., L.R.C.S. Edin., has been appointed Casualty Officer; and Miss Louisa Woodcock, M.D. Lond., B.S., has been appointed Clinical Assistant.

A Reuter's telegram from Rio de Janeiro of July 17 stated that an announcement had been made that day by Dr. Oswaldo Cruz, Director-General of the Sanitary Service, at the Rio de Janeiro Academy of Medicine to the effect that the bacillus of smallpox has been discovered in the course of bacteriological researches carried out at the Oswaldo Cruz Institute by Drs. Henrique Beaurepaire de Aragao and Prowazek.

At a meeting of the Manchester Infirmary Board on July 13, the following letter addressed to Sir William Cobbett, Chairman of the Board of Management, the Royal Infirmary, Manchester, from the Right Hon. R. B. Haldane, was read : "I am commanded by the King to express to you the genuine pleasure which the inspection of the Royal Infirmary this afternoon gave to himself and to the Queen. The admirable scientific organisation, the well-considered planning of the building, and the evidences of a high standard of medical and surgical attainment in the staff gave their Majesties the sense that they had visited an institution of which Manchester may well feel proud."

The eleventh South African Medical Congress will be held in Durban, August 2 to 7, 1909. His Excellency Sir Matthew Nathan, K.C.M.G., R.E., the Governor of Natal, will open the proceedings on Monday August 2. The President of the Congress is Dr. H. A. Dumat, and the Vice-Presidents Drs. W. Watkins-Pitchford and W. J. Hill. The Hon. Secretary is Dr. P. Murison, and the Hon. Treasurer Dr. G. L. Bonnar. The work of the Congress will be divided among six sections, which are respectively Medicine, Surgery, Obstetrics and Gynæcology, Public Health, Ophthalmology, and the other special subjects grouped together.

### THE BRITISH MEDICAL ASSOCIATION.

The agenda published for the Annual Representative Meeting of the British Medical Association, due at Belfast on July 23, contained a supplementary report dealing with matters which have arisen since the Annual Report was published. The suggested recognition of a special class of "consultants" is favourably considered in a special report upon this question by the Central Ethical Committee. The Committee suggest that such a class, distinguished by the fact that they confine their practice to the treatment of patients in co-operation with other practitioners, would be in the best interests of the profession. Some such definition of the scope of the work of the two chief branches of the profession would, in their opinion, tend greatly to reduce friction and jealousy between medical men; and, by promoting harmony and co-operation within its ranks, would advance the honour and interest of the profession. They recommend that their report should be referred to the Divisions with a request that each Division will express an opinion upon this proposed recognition of a special class of consultants on the lines indicated; and that each Division, if favourable to the main proposal, will further agree to the drawing up by the British Medical Association of regulations for the conduct of the practice of such a class.

Princess Christian and Princess Victoria of Schleswig-Holstein attended the "View Day" of the Frimley Sanatorium and Convalescent Home attached to the Brompton Hospital for Consumption on Saturday last, July 17. The Sanatorium was opened five years ago by the Prince and Princess of Wales, and the building contains 150 beds, of which 104 are for men, 42 for women, and four for young children, all now occupied. Besides these, 150 ex-patients, most of them in excellent health, were present. The Princesses were received by General Lord Cheylesmore (Chairman of the Committee), Mr. St. Croix Rose (Vice-Chairman), Dr. Robert McGuire (Chairman of the Medical Committee), Dr. M. S. Paterson (Medical Superintendent of the Sanatorium), and Mrs. Paterson, Miss Lloyd-Still (Lady Superintendent of the Brompton Hospital), and Mr. Frederick Wood (Secretary), who conducted the Royal visitors through the wards and afterwards through the grounds, in which, among other useful works, a concrete reservoir has been constructed entirely by the male patients, by whom the whole of the grounds are kept in order, with the result that no money is spent on labour at the Sanatorium.

### ARRANGEMENTS FOR THE ANNUAL MEETING OF THE BRITISH MEDICAL ASSOCIATION AT BELFAST.

The seventy-seventh annual meeting of the British Medical Association is just commencing at Belfast as we go to press, and arranged for Friday, July 23, and the following six week-days. Sir William Whitla, Professor of Materia Medica and Therapeutics, Queen's College, Belfast, is the distinguished President of this year's Congress.

Fifteen Scientific Sections have been arranged, and will meet daily from Wednesday to Friday in the Queen's College at 10 A.M., namely : Anatomy and Physiology, Dermatology and Electro-Therapeutics, Hæmatology and Vaccine Therapy, Diseases of Children, Navy, Army and Ambulance, Hygiene and Public Health, Laryngology, Otology and Rhinology, Medicine, Obstetrics and Gynæcology, Ophthalmology, Pathology, Pharmacology and Therapeutics, Psychological Medicine, Surgery, and Tropical Medicine. The programmes throughout promise particularly interesting discussion, owing to the large attendance of men of eminence from foreign countries and various parts of the British Empire.

On Tuesday next, July 27, at 2.30, Sir William Whitla will be inducted to the Presidential Chair, while at 8.30 P.M. that evening he will deliver his Presidential Address in the Assembly Hall, Belfast. On Wednesday, July 28, at 2.30 P.M., Dr. R. W. Philip, F.R.C.P.Edin., will deliver an address on Medicine in the Library, Queen's College. On Thursday, July 29, at 12.30 P.M., the address on Surgery will be delivered in the Library, Queen's College, by Mr. Edward James Barker, F.R.C.S. On Friday, July 30, at 12.30 P.M., Sir John W. Byers, M.D., will deliver an address on Obstetrics in the Library, Queen's College, while at 8 P.M. a popular lecture will be delivered by Dr. J. A. Macdonald. Finally, at 8.30 o'clock of the same evening, the termination of the Congress will be celebrated officially by a Reception, in the Botanic Gardens, of the assembled members, at the invitation of the President and local members of the Ulster Branch.

We are indebted to the Secretary of the British Medical Association for his courtesy in forwarding us the above particulars of special items in the programme arranged for the present meeting of the Association. We anticipate an unqualified success for this year's gathering at Belfast.

